# PCSK9 inhibitor failure in a statin-intolerant FH patient with a novel LDLR variant: a case report

**DOI:** 10.3389/fcvm.2025.1644145

**Published:** 2025-09-22

**Authors:** Yuan Li, Huiyan Jiang, Yajuan Xiong, Simin Yan, Xin Chen

**Affiliations:** ^1^ Department of Pharmacy, Nanjing Drum Tower Hospital, Affiliated Hospital of Medical School, Nanjing University, Nanjing, Jiangsu, China; ^2^ Department of Cardiovascular Medicine, Nanjing Drum Tower Hospital, Affiliated Hospital of Medical School, Nanjing University, Nanjing, Jiangsu, China

**Keywords:** familial hypercholesterolemia, low-density lipoprotein receptor (LDLR), PCSK9 inhibitor, gene mutation, statin intolerance, case report

## Abstract

**Background:**

Approximately 3.8 million patients in China suffer from familial hypercholesterolemia (FH). Statins and PCSK9 inhibitors are recommended by guidelines as therapeutic agents. Nevertheless, cases in which patients demonstrate statin intolerance and an abnormal response to PCSK9 inhibitors present a significant challenge to the clinical treatment of the condition.

**Case presentation:**

We report a 56-year-old Chinese female diagnosed with heterozygous familial hypercholesterolemia (HeFH). After taking simvastatin, she had elevated transaminases and creatine kinase levels, leading to a transition to PCSK9 inhibitor therapy. Unfortunately, the patient exhibited an absence of the desired response to three different PCSK9 inhibitors. A novel heterozygous missense variant in the LDLR gene (exon 11, c.1700C > T, p.Thr567Ile) was identified through related gene sequencing and genetic testing also revealed a heterozygous variant in the HTR7 gene. In light of the findings, she was treated with a combination of rosuvastatin and ezetimibe. This treatment resulted in the achievement of target lipid levels. During the follow - up, no adverse events were reported.

**Conclusion:**

The study highlights that genetic testing should be considered for FH patients who experience failure with PCSK9 inhibitors, as novel LDLR variants may account for resistance and inform personalized treatment.

## 1 Introduction

FH is an inherited disorder of cholesterol metabolism that results in elevated levels of low-density lipoprotein cholesterol (LDL-C) in the blood. The clinical manifestations of patients are primarily determined by their genotype and exhibit phenotypic heterogeneity (1). Typically, the hereditary condition known as FH is classified into two main types, such as autosomal dominant hypercholesterolemia (ADH) and autosomal recessive hypercholesterolemia (ARH). A patient with ADH has one affected parent and often presents with a positive family history of premature coronary heart disease (CHD). The etiology of ARH is attributed to pathogenic variants in the LDL receptor adaptor protein 1 (LDLRAP1) gene, with the condition manifesting in early childhood (2). In the case of heterozygous individuals or even in homozygous population with altering mutations like the p.(ala431thr) (3), lower levels of LDL-C and milder phenotypes are exhibited in comparison to those with homozygous familial hypercholesterolemia (HoFH). This frequently results in cases being overlooked (1).

The management of FH primarily encompasses dietary and pharmacological interventions (4). Statins and proprotein convertase subtilisin/kexin type 9 (PCSK9) inhibitors are considered the therapeutic options for these patients. However, studies (5–8) have indicated that Asian populations exhibit a comparatively poorer tolerance to statins. On the other hand, it should be noted that not all individuals with FH respond positively to PCSK9 inhibitors (9, 10). Thus, establishing appropriate diagnostic and treatment protocols and elucidating its mechanisms is essential to assure goal attainment for these patients. To the best of our knowledge, there are no case reports of patients with FH for whom all three PCSK9 inhibitors have been ineffective. In this study, we present a case of statin-intolerant HeFH that exhibited an abnormal response to three PCSK9 inhibitors.

## 2 Case presentation

### 2.1 Patient information and clinical findings

In April 2021, a 55-year-old female patient was first found to have elevated blood lipids during a health checkup. Notwithstanding this, she did not undergo any lipid-lowering treatment immediately. In March 2022, this patient attended the Outpatient Lipid Management Department due to hyperlipidaemia. Laboratory analysis revealed that the subject's Total Cholesterol (TC) levels were 9.01 mmol/L (normal range: 5.00–5.71 mmol/L), LDL-C levels were 6.09 mmol/L (normal range: <3.4 mmol/L), and lipoprotein(a) [Lp(a)] levels were 794 mg/L (normal range: 1–300 mg/L). The patient exhibited no history of chronic diseases and was not currently taking any medication. The patient's physical examination results are outlined below: heart rate of 65 beats per minute, blood pressure of 123/81mmHg and all markers of myocardial injury, glycosylated hemoglobin, thyroid function and liver and kidney function tests were all within the normal reference range. On the same day, the patient underwent a colour Doppler ultrasound examination of the bilateral carotid arteries. This examination revealed symmetrical diameters of the bilateral carotid arteries, normal intima-media thickness (right IMT: 0.08 cm, left IMT: 0.08 cm), adequate blood flow filling in each segment of the carotid arteries, and normal blood flow velocity and spectral morphology. The patient has two siblings, specifically an older brother and a younger sister. A lipid profile test revealed that the patient's older brother had TC levels exceeding 7.5 mmol/L at the age of 50. To date, the patient's younger sister has exhibited no anomalies in her lipid profile. According to the Simon Broome criteria (11), the patient was diagnosed with possible FH at that time.

### 2.2 Therapeutic intervention

**Figure 1:**
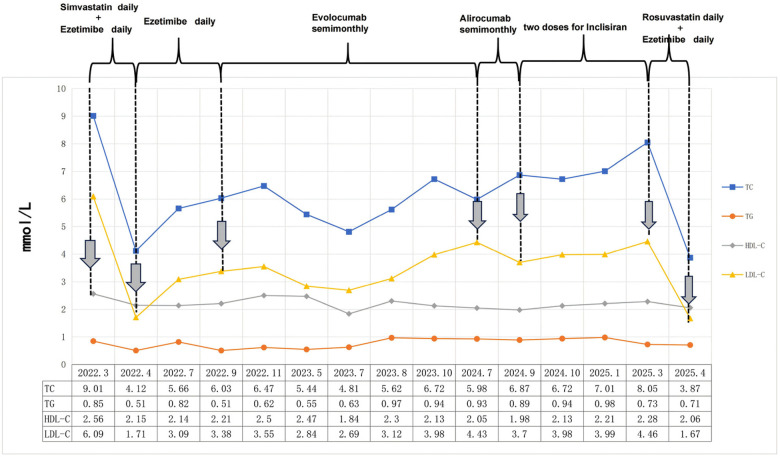
The lipid levels of this patient were monitored over a 3-year follow-up period, and the values are reported in mmol/L.

Firstly, the physician prescribed simvastatin (40 mg, administered once daily) and ezetimibe (10 mg, administered once daily). Following this, a long-term follow-up evaluation of the patient's blood lipid levels was conducted, and a waveform chart was generated based on these levels and the lipid-lowering treatment strategies employed (Figure 1). During subsequent follow-ups, symptoms of intolerance to simvastatin manifested, as indicated by elevated levels of ALT at 64.5 U/L (normal range: 5–40 U/L) and CK at 833 U/L (normal range: 20–174 U/L) concomitant with muscle discomfort. The patient was confirmed to have statin intolerance, and treatment with ezetimibe alone was initiated; however, the patient's lipid levels continued to fall short of the desired target. Two compliance assessments of the patient were conducted using the Morisky Medication Adherence Scale (MMAS) (12) in July and September of 2022, achieving scores of 6 and 7, respectively. These scores suggest a potential for nonadherence to medication. The patient also reported experiencing inadequate exercise and dietary control during this period due to family circumstances.

In September 2022, the therapeutic approach aimed at reducing serum cholesterol levels was transitioned to evolocumab (140 mg twice a month). Nevertheless, over the course of the subsequent ten months, the patient's blood lipid levels initially demonstrated a decline, subsequently followed by an uptick. After that, the treatment regimen was modified to alirocumab, administered at a dosage of 75 mg once every two weeks. After a period of three months during which the aforementioned therapy was administered, the patient's LDL-C level exhibited a decline of only 16.4%. It is acknowledged that the reasons for the abnormal response to PCSK9 inhibitors may include the presence of anti-drug antibodies, increased *in vivo* PCSK9 concentration, and other factors (13). However, it is regrettable to note that the necessary equipment to detect blood PCSK9 concentration is not currently available at this hospital. Furthermore, the patient declined both genetic testing and other statin medication therapies at that time. Consequently, the decision was taken to administer inclisiran in order to treat the dyslipidaemia. Despite the administration of two standardized doses of inclisiran, the patient's blood lipid levels remained inadequately controlled. Following a comprehensive evaluation of the patient's case, a treatment regimen including rosuvastatin (20 mg daily) and ezetimibe (10 mg daily) was administered. This therapeutic approach resulted in a substantial reduction in blood lipid levels, thereby achieving the desired therapeutic objective. During the subsequent follow-up period, there were no reports of any untoward occurrences.

### 2.3 Genetic analysis

In March 2025, following the procurement of the patient's consent, genetic testing was conducted to provide further clarification on the diagnosis. A novel heterozygous missense variant in the LDLR gene (exon 11, c.1700C > T, p.Thr567Ile) (Table 1) was identified by gene sequencing. In light of the findings, the patient was diagnosed with HeFH in accordance with the Simon Broome criteria (11) and the diagnostic considerations established by the American Heart Association (14). In this patient, gene sequencing revealed a heterozygous variant in the HTR7 gene (intron1, c.539 + 22547G > A, p.?)(Table 1). Unfortunately, the patient's parents passed away in an accident many years ago, and the siblings of our patient refused to carry out genetic testing.

**Table 1:** Single nucleotide variants and their associated phenotypes.

Gene	Chromosomal location	Transcript Exon	Nucleotide Amino acid	Genotype	Phenotype
LDL-R	chr19:11226883	NM_000527.5; exon11	c.1700C > T (p.Thr567Ile)	Het	HeFH
HTR7	chr10:92594343	NM_019859.4; intron1	c.539 + 22547G > A (P.?)	Het	Statin-induced myopathy

## 3 Discussion

Statins and PCSK9 inhibitors are recommended as lipid-lowering therapies for FH patients (15). However, in this case, elevated levels of transaminases and creatine kinase were observed in the patient during the administration of simvastatin. Over the subsequent 30 months, the patient sequentially utilized three different PCSK9 inhibitors, yet no significant lipid-lowering effect was observed. This presented a challenge in determining the clinical lipid-lowering programmes.

Several studies (16–18) have indicated that the usage rate of statins among the Chinese population is significantly lower than that observed in developed countries. This disparity may be attributed to patients' concerns regarding adverse reactions to statins. The most common adverse reactions to statins are muscle-related adverse reactions and liver dysfunction (18). The genes of ATP-binding cassette transporter B1 (ABCB1) (19), solute carrier organic anion transporter family member 1B1 (SLCO1B1) (20), the cytochrome P450 (CYP) family (21) and 5-hydroxytryptamine (serotonin) receptor 7 (HTR7) (22) have been demonstrated to be associated with statin adverse drug reactions (ADRs).

In this patient, gene sequencing revealed a heterozygous variant in the HTR7 gene (intron1, c.539 + 22547G > A, p.?) (Table 1). Genomic research reveals a statistically significant association between simvastatin-induced myalgia and the rs1935349 variant in the HTR7 gene (22). This statin-specific association explains SAMS occurring in this patient after taking simvastatin. Additionally, it was noted that the incidence of myopathy is much more with simvastation (50%) than with other statins like rosuvastatin (10.8%) (23). In accordance with the results of genetic testing for antihyperlipidaemia medication, the patient was administered rosuvastatin and ezetimibe treatment without any reported adverse reactions.

In consideration of the inadequate compliance with statin therapy demonstrated by patients (24), it can be hypothesised that PCSK9 inhibitors may possess significant therapeutic potential in China. Alirocumab and Evolocumab are fully human monoclonal antibodies that target PCSK9, which were found to be effective and safe in reducing LDL-C (25–27). On 22 August 2023, inclisiran was launched in China. The drug has been demonstrated to target and disrupt the synthesis of liver PCSK9 protein via an intracellular RNA interference mechanism, thereby upregulating the expression of LDL receptors and decreasing LDL-C levels (28). As the world's first siRNA ultra-long-acting lipid-lowering pharmaceutical agent, study (29) in Asian populations has indicated that 71.7% of participants who received 300 mg of inclisiran sodium experienced a reduction in LDL-C of ≥50% by day 330, compared to just 1.5% with placebo. However, it is important to note that a recent study (13) demonstrated that PCSK9 inhibitors exhibited an abnormal response rate of 13.1% in real-world settings, with nearly half of these cases attributable to inadequate adherence. Consequently, the initial step in assessing the unresponsiveness of PCSK9 inhibitors involves monitoring medication adherence. In this case, following treatment with three distinct PCSK9 inhibitors, the patient exhibited an average LDL-C level of 3.67 mmol/L. In view of the fact that all the injections were administered by the nurses, it is imperative to undertake a more thorough analysis in order to ascertain the underlying causes.

Secondly, the availability of PCSK9 assays has the potential to assist clinicians in more effectively diagnosing abnormal responses (30). However, economic factors have been a significant barrier to the implementation of this technology. As indicated by Bruce A. Warden (13), the prevalence of hereditary hemochromatosis (HeFH) was found to be higher among subjects demonstrating unusual responses. Additionally, case reports (30, 31) also support the notion that it is crucial to implement a comprehensive screening process for HeFH and to identify the relevant gene sequences.

A recent study (32) has revealed that LDLR constitutes the primary pathway through which the body eliminates LDL-C, and mutations that affect its conformational changes confer resistance to both PCSK9 and its inhibitors. The LDLR gene (MIM Number: 606945) consists of 18 exons, which are associated with the functional domains of the LDLR protein. In this patient, gene sequencing revealed a novel heterozygous missense variant in the LDLR gene (exon 11, c.1700C > T, p.Thr567Ile). The variant was absent from the control databases of the Exome Sequencing Project, the 1,000 Genomes Project, the Exome Aggregation Consortium and the GnomAD (Genome Aggregation Database). REVEL software predicts that the variant is damage. The results of the functional study on the adjacent site c.1694G > T (p.G565V) provide the basis for the following hypothesis: that the mutation in question represents a missense mutation within the *β*-propeller domain and may be classified as a class 2 mutation. LDLR binds to apoB100 through two independent interfaces (BS1 and BS2), with BS2 being formed by the β-propeller domain of LDLR binding to the N-terminal domain of apoB100 (33). This facilitates the interaction between LDL-C and LDLR, thereby promoting cellular uptake of LDL-C absorption. Consequently, we further deduce that this patient's elevated LDL-C levels result from inherent LDL-R functional defects, independent of circulating PCSK9 concentrations.

In addition to this, the study (34) suggests that a range of LDLR mutation phenotypes exhibit varied responses to lipid-lowering therapies. It is noteworthy that in patients exhibiting class 2 LDLR variants, statins demonstrated a heightened efficacy in reducing blood lipid levels when compared to those with class 5 LDLR variants (35). During the follow-up period, it was observed that the patient taking simvastatin and rosuvastatin exhibited a reduction in LDL-C levels (71.92% and 62.56%, respectively), demonstrating significant lipid-lowering effects.

It is important to consider several limitations when interpreting the findings of this study. Firstly, as it is a case report from a single center, it may not be representative of the general population. Secondly, the genetic origin of this variant remains to be elucidated due to the absence of parental DNA. Thirdly, further studies are required to elucidate the potential pathogenicity and expression mechanisms of mutated genes, which would help to further understand the structure of LDL and its interaction with LDLR.

## 4 Conclusion

In this case, genetic testing yielded pivotal insights for an FH patient afflicted with statin intolerance, leading to the identification of the rs1935349 variant in the HTR7 gene. This finding prompted a change to alternative statin regimens, which were shown to successfully avoid adverse drug reactions. On the other hand, genetic testing revealed a novel missense variant in the LDLR gene in this patient, a discovery of significant importance that filled the gaps in the existing gene databases. In summary, genetic testing should be considered for FH patients who experience failure with PCSK9 inhibitors, as novel LDLR variants may account for resistance and inform personalized treatment.

## Data Availability

The datasets presented in this study can be found in online repositories. The names of the repository/repositories and accession number(s) can be found in the article/Supplementary Material.
